# Assessment of Parental Perceptions of Socio-Psychological Factors, Unmet Dental Needs, and Barriers to Utilise Oral Health Care in Autistic Children

**DOI:** 10.7759/cureus.27950

**Published:** 2022-08-12

**Authors:** Ankita Verma, Harsh Priyank, Butta Viswanath, Jaimesh Kumar Bhagat, Saurav Purbay, Mahalakshmi V, Sahana Shivakumar

**Affiliations:** 1 Department of Pedodontics and Preventive Dentistry, Hazaribagh College of Dental Sciences and Hospital, Hazaribagh, IND; 2 Department of Conservative, Endodontics & Aesthetic Dentistry, Dental College, Rajendra Institute of Medical Sciences, Ranchi, IND; 3 Department of Conservative, Endodontics and Aesthetic Dentistry, Dental College, Rajendra Institute of Medical Sciences, Ranchi, IND; 4 Department of Conservative Dentistry and Endodontics, Mithila Minority Dental College and Hospital, Darbhanga, IND; 5 Department of Conservative Dentistry and Endodontics, Hazaribagh College of Dental Sciences and Hospital, Hazaribagh, IND; 6 Dentistry, Sub Divisional Hospital, Bermo, IND; 7 Public Health Dentistry, Peoples College of Dental Sciences and Research Centre, Bhopal, IND

**Keywords:** questionnaire, unmet need, oral hygeine status, dental caries, perception, validity, parents, health care service, austism

## Abstract

Introduction: Parents' participation is crucial in the dental health intervention of children with autism spectrum disorder (ASD). Parenting children with ASD is extremely stressful and challenging. Parents and other caregivers have a responsibility to care for and raise children with ASD. Parental perception of the condition, situational adaptation, and attitude towards the issue are significant indicators of how they will respond and eventually adapt to it.

Methodology: A comparative, descriptive study was done on parents of 154 autistic children and 235 normal children. An 11-variable questionnaire eliciting various details of socio-psychological factors affecting the utilisation of oral health care was designed and validated. The questionnaire also included parameters regarding barriers to accessing dental services. The dental caries and oral hygiene status of all children were examined using the Basic Oral Health Survey 2013 proforma. Chi-square and independent t-tests were applied to find significant differences between the groups.

Results: A greater number of male children (61.0%) were observed among autistic children. Parental perception regarding socio-psychological concerns with raising an autistic child showed significant differences for all variables between the groups except for assistance in the child’s task with 90.2% as against 55% of the parents with non-autistic children. Parents of autistic children were more receptive to the idea of focus group discussion. Decayed teeth were significantly found to be higher in autistic cohorts (2.554 ± 1.616 versus 1.779 ± 1.841). Oral hygiene status was also statistically significant amongst autism-affected children than those who were not at p =0.000.

Conclusion: The present study explored various socio-psychological factors of parental perception of autistic children. A better outcome can be suggested when parents gained awareness regarding various strategies and treatment options available for their child's oral health. Furthermore, dental health can be improved by bringing in certain environmental modifications in which the autistic child is groomed.

## Introduction

Autism was first described in 1943 by Leo Kanner, a child psychiatrist, as a neurodevelopmental condition [1]. It is characterised by having difficulties in interaction, communication issues and exhibiting repetitious behaviour. In recent years, the prevalence of autism has grown significantly [2]. Literature data have reported a prevalence of one amongst 68 children affected with this condition at the tender age of eight years [2]. This implies that there is an escalating percentage of dentists facing autistic children in their careers. As a result, there is a greater need to increase awareness to create dental strategies that are more appropriate for people with autism spectrum disorder (ASD), and a greater demand for research in this area throwing light on every aspect. 

A consistent relationship is established between social capital (social capital refers to the network of relationships amongst people who live and work in a particular society, enabling that society to function effectively) and health perceived by earlier social ecology studies [3,4]. Research has also revealed that social support, a component of the social environment, is a significant predictor of an individual's subjective mental health [5,6]. In addition, parents' evaluations of their own personal traits, such as their mental health and parenting skills, may also affect how they view their kids in general [7]. However, little research has focused on specific social and psychological elements that can affect parents' assessments of their child's autism symptoms.

Families with an ASD child face a wide range of challenges, such as reduced parenting competence, higher stress levels, issues with mental and physical health, severe economic hardship, time constraints, issues with sibling adjustment, limited social support, and family discord [8]. Literature evidence has suggested that ASD affects families more significantly as compared to other conditions [9]. However, research in this field is more pronounced in the developed countries with subsequent appropriate interventions planned. The same cannot be said for the developing countries. This has prompted several authors to advocate for furthering research on ASD from a global perspective [10,11]. Raising children with ASD can overwhelm parents and families in both developed and developing nations, more pronounced in the latter. In industrialised nations, extensive professional support services for parents and children with ASD by trained professionals and a standardised education system are the norms; however, these programmes are almost non-existent in developing nations such as India, and relatively few ASD children have access to effective interventions. Furthermore, there is an accumulation of unmet dental needs in this population, possibly because of difficulty in accessing oral health care. Hence, the present study was undertaken to answer the following research statements “What is the socio-psychological perception of a parent raising an autistic child?”, “What are the unmet dental needs of an autistic child?” and “What are the possible barriers in utilising dental health care ?”.

## Materials and methods

A cross-sectional study was conducted to obtain data regarding parental perception. Parents of autistic children were recruited from two special schools in the city. One parent of an autistic child was interviewed, accounting for a sample of 154 parents. Considering that mothers spend most of their time with the children attending to their needs and grooming them, the parent interviewed was the mother. Along with this, a convenient sample of 235 parents of their normal counterparts was also assessed, recruited from schools in the neighbourhood. Ethical consent to carry out the study was obtained from the Institutional Ethical Committee of Peoples College of Dental Sciences and Research Centre, Bhopal, India (EC202104). Permission from school authorities was taken for conducting the interview of parents and oral examination of children. The confidentiality of every individual was strictly maintained. A letter was sent to each autistic child's parent describing the objectives of the study in detail and to obtain their willingness to participate. If the parents were willing, a suitable time was fixed wherein the parent could spare some quality time for the interviewer. Consenting parents were interviewed at the time of pick-up after school. Oral examination of all children was done in their classroom itself. The examination was done by a calibrated examiner using a mouth mirror and explorer under adequate illumination. Parents of normal children were interviewed on the day of the parents-teacher meeting after obtaining prior permission by a note sent in the child's diary. 

Scrutiny of medical records was made with prior permission from the school authorities to ensure strict eligibility criteria for consenting individuals. Parents of children with known neurological diseases or those exhibiting signs of focal neurology, sensory impairments such as blind, deaf and dumb, and known history of epilepsy and head injury were excluded.

A questionnaire was framed to elicit various social and psychological factors assessing perception by parents of an autistic child and their barriers to utilising dental care as the authors could not find any in the literature. The instrument also recorded various barriers to the utilisation of dental health services as felt by the parents of their wards. 

Validity of the questionnaire: A ten-variable questionnaire was prepared by the investigators. The convergent validity of the items in the questionnaire was evaluated using Pearson’s correlation coefficient. The obtained value was greater than the critical value for all questions and was highly significant, and hence, every question was considered to be a valid one. A two-team expert panel was set up to check for the degree of relevance. Item-Content Validity Index (I-CVI) for all the items scored “1” to achieve universal or cent percent agreement. Scale level-Content Validity Index (S-CVI) was based on the summary of all the I-CVI scored 0.9 making it a relevant questionnaire.

Before the start of the study, the questionnaire was pilot tested on 10 parents to check for their feasibility and those who were not included in the study.

Data analysis: The collected responses were analysed using Statistical Package for Social sciences (SPSS version 23.0; IBM, Armonk, NY). The chi-square test of association was applied to find differences between parental perception of autistic children and normal children. An Independent "t"-test was run to find significant differences in the decayed teeth and oral hygiene status between autistic children and normal children. A p-value less than 0.05 was considered to be statistically significant.

## Results

A total of 154 autistic children and 235 non-autistic children completed the study. The socio-demographic data of the population are summarised in Table 1. Clearly, a male predominance was noted, with 94 (61.0%) being males. None of the children were of the "only child” category of family. Three out of 154 autistic children had siblings with autistic traits, while it was none among their normal counterparts.

**Table 1:** Descriptive data showing characteristics of autistic children and their mothers Values represented are n (%).

Characteristics (autistic children)	Values
Gender	
Male	94 (61)
Female	60 (39)
Age (in years) (mean ± SD)	10.130 ± 2.324
Single child	
Yes	0 (0.0)
No	154 (100.0)
First child	
Yes	112 (72.7)
No	42 (27.3)
Siblings with autistic characteristics	
Yes	3 (2.0)
No	151 (98.0)
Maternal age	
<35 years	85 (55.1)
>35 years	69 (44.9)

Parental perception regarding issues with raising an autistic child showed significant differences for all variables at p <0.0001 as compared to non-autistic children as seen in Table 2. Incompetency in the existing educational system to help their child in coping with learning and vocational opportunities was the most commonly expressed concern in 79.2% of parents of autistic wards as against 31% of the normal children’s parents. 

**Table 2:** Distribution of parental perception variables in raising ASD-affected children and non-autistic children * = Significant; NS = Not Significant. ASD, autism spectrum disorder.

	Autistic children (n = 154)		Non-autistic children (n = 235)		Chi-square statistic	p-Value
	N	%	N	%		
Do you think that raising a child with ASD has a greater financial burden?						
Yes	115	74.6	38	83.8	133.4512	<0.00001*
No	39	25.4	197	16.2		
Are you mentally stressed and experience anxiety in caring for an ASD-affected child						
Yes	107	69.4	114	48.5	16.673	0.000*
No	47	30.6	121	51.5		
Is your quality of life affected negatively in any way being a parent?						
Yes	95	61.6	104	44.2	11.315	0.0007*
No	59	38.4	131	55.8		
Do you feel health care and oral healthcare services available are adequate in catering to your child’s need?						
Yes	103	66.8	82	34.8	61.782	<0.00001*
No	51	33.2	153	65.2		
Do you feel educational system prevailing can help/facilitate your child?						
Yes	122	79.2	73	31.0	86.301	<0.00001*
No	32	20.8	162	69.0		
Have you experienced any episode of discrimination or social stigma as a parent?						
Yes	104	67.5	27	11.4	118.861	<0.00001*
No	50	32.5	208	88.6		
Has safety concerns stopped you from allowing your child to sport or cultural activities?						
Yes	98	63.3	56	23.8	70.5747	<0.00001*
No	56	36.7	179	76.2		
Do you find it difficult in assisting your child to complete tasks?						
Yes	41	26.6	57	24.2	0.2768	0.598 (NS)
No	113	73.4	178	75.8		
Do you feel apprehensive to change from the routine of your child?						
Yes	93	60.3	126	81.8	1.7344	0.187 (NS)
No	61	39.7	109	18.2		
Do you find it annoying when others give opinion about your child?						
Yes	80	51.9	94	61.0	5.3721	0.02*
No	74	48.1	141	39.0		
Do you welcome peer group/focus discussion to facilitate communication with the child?						
Yes	139	90.2	85	55.1	111.437	<0.00001*
No	15	9.8	150	44.9		

In comparing the autistic children and the normal child population, it was noted that a clear distinction was found with unmet needs to be higher in the former in than the latter group. Filled teeth (met needs) were higher in normal children with a mean of 1.844 ± 0.168 as compared to 0.994 ± 0.136 in the other group, which was statistically significant as seen in Table 3.

**Table 3:** Comparative assessment of dental health status between autistic and non-autistic children * = Significant. dmft, decayed, missing and filled teeth.

Variable	Autistic children (mean ± SD)	Non-autistic children (mean ± SD)	t-Test statistic	p-Value
Mean dmft	2.987 ± 0.150	2.254 ± 0.145	12.118	0.000*
Decayed teeth	2.554 ± 1.616	1.779 ± 1.841	2.366	0.020*
Filled teeth	0.994 ± 0.136	1.844 ± 0.168	21.434	0.000*
Oral hygiene index simplified	3.559 ± 0.360	2.829 ± 0.262	18.366	0.000*

Table 4 delineates the various barriers perceived by parents in meeting their child’s needs. The greatest limitation to access dental health services as perceived by the parents was timings of appointments (77.2%) followed by issues with waiting periods in 66.8% of the study population. Fear of pain for dental treatment was almost equally present in both groups of parents, which was non-significant at p =0.321.

**Table 4:** ​​​​​​​Barriers for utilisation of oral health care among autistic and non-autistic children * = Significant; NS = Not Significant.

Barriers	Autistic N (%)	Non-autistic N (%)	Chi-square statistic	p-Value
High cost	85 (55.1)	103 (43.8)	4.812	0.028*
Transportation to dental clinic	92 (59.7)	58 (24.6)	46.799	<0.00001*
Timings of appointment	119 (77.2)	76 (32.3)	75.131	<0.00001*
Problems with waiting periods	103 (66.8)	83 (35.3)	37.146	<0.00001*
Sight of dental unit and working space	88 (57.1)	91 (38.7)	12.7063	0.0003*
Fear of pain	74 (48.0)	125 (53.1)	0.9835	0.321 (NS)
Comprehension difficulties	81 (52.5)	66 (28.0)	23.777	<0.00001*

## Discussion

The present cross-sectional study assessed the socio-psychological factors of parents grooming autistic children and compared them with that of parents raising normal children. It was observed that the proportion of male children was more compared to females in this study. This finding is comparable to that of Wilson CE et al., who found that male ASD patients were more prevalent than female ASD patients, and to the findings of the American Autism Association, which said that male ASD prevalence was 4:1 [12,13]. The notion of boys carrying over the family tree in the Indian subcontinent might have pushed the parent for preferential treatment.

The financial burden was found to be the greatest amongst all other variables perceived in 74.6% which was statistically significant. Concurrent results were found in the studies of Al Jabery et al., Hoefman et al. and Dababnah et al., who also reported that financial incompetencies prevented families from seeking care for their children. The absence of publicly funded schemes and programs for autistic children in our country further adds to the finance load [14,15]. Mental stress was another factor perceived greatly by the parents. Studies by Benson and Karlof, Frye et al. and Hock et al. support this finding [16-18]. It was also noted that the coping skills of these parents were less adaptive as compared to the normal child's parents. Lack of social and emotional support for families appears to be a common problem. It has been noted that even in industrialised countries, parents of children with ASD are dissatisfied with or disappointed with the level of assistance provided to their children [19,20].

The oral health conditions of autistic children were definitely more compromised, which could possibly be because of a lack of manual dexterity and various barriers associated with utilising oral care as presented in our results. The educational system for autistic children needs special mention. Typically, autistic students attend special needs schools if they are diagnosed at earlier stages. They may be attending along with children posing intellectual disabilities, cerebral palsy and hearing loss, which might not be a suitable educational setting for them. Only a small percentage of autistic students attend regular public schools in special needs classrooms. The quality of the education provided is inconsistent across the board. Standards are not being kept under any kind of watch. Most institutions, even those specialising in autism, lack staff who are educated in behavioural principles or autism awareness [21].

There is an unknown number of children with autism in ordinary classrooms. Undiagnosed children make up a bigger percentage of this population. A few children might overcome this with few difficulties. However, as kids grow older and the educational system becomes more rigid and inflexible, many children who are enrolled in conventional schools are compelled to leave. In addition to the increasing academic pressure, many students drop out of school due to their inability to cope with bullying and social exclusion. As a result, parents are at crossroads whether or not to inform their child's teacher and school of his or her diagnosis of autism. Consequently, the social stigma has resulted in the mounting of autism prevalence.

Our study's findings are consistent with those of Hock R et al., who found that parents of children with ASD have worse mental health and coping skills than other parents, as well as higher levels of annoyance [22]. Additionally, they reported lower relationship satisfaction and neighbourhood social capital, which may indicate that these parents feel more alone in their neighbourhoods and less supported at home. Focus groups are becoming more and more common in health and medical research and are strongly recommended for autistic children to improve their behaviour. It is a method of getting information from parents of children with ASD about their feelings, views and opinions concerning the condition in an individual and societal context through a moderator-led discussion. Parents of children with ASD have benefitted immensely from it [23].

Families residing closer to urban areas and college towns may have a higher chance of receiving a thorough autism evaluation from a specialist [24]. However, it is imperative that doctors will depend heavily on parents' perceptions of their child's problems to arrive at a diagnosis in the absence of such an evaluation. In either case, a health professional's overall diagnosis and supervision of a child with ASD include at least some consideration of parents' perceptions [25]. The frequent and regular use of parent-completed rating scales and screening instruments in the diagnosis of ASD provides evidence for this. Professionals can be guided in their appraisals of the circumstances around the child and family by an understanding of these domains. For instance, doctors may be able to construct a more detailed picture of the reciprocal relationship between the child's symptoms and parent functioning if they are aware of parents' annoyance and mental health. This knowledge could further help professionals create family-centred therapy strategies. The current study extends the theoretical understanding of socio-psychological perceptions of parents towards oral health by parents. It has an advantage in including all domains of the social and psychological framework. Additionally, this research establishes a basis for future investigations further into causality between social environment, the psychological set-up, and perceptions of the health of parents with ASD-affected children. 

Using self-reported data has the potential to introduce bias, especially when it comes to questions on how individuals perceive their own behaviours, where issues of social desirability might play a role in how they answer those questions. Because there are no other simple means to acquire information on social and psychological perceptions, this is the only method to be used. Owing to the cross-sectional design of the research, a directional causal association cannot be achieved between the variables. Some researchers have maintained that parent apprehension is directly related to the severity of ASD symptoms. However, it could also be vice versa, wherein parents’ apprehension made the parents to perceive their child’s symptoms to be severe. A drawback of the study is the two response choices to gauge parent perception. This might not be sensitive enough to capture the severity of perception as the responses were of the absolute, dichotomous type, that is either yes or no. 

## Conclusions

The study results suggested social and psychological factors as perceived by parents of autistic children did affect the utilisation of dental health services. Unmet dental needs were also higher in the autistic wards. Parents' perception of autism and its associated factors may also influence how they interact with professionals, how they see their kids, their perception of duties or responsibilities regarding their child's conditions, and the kind of intervention they favour. Professionals can give better interventions for children with ASD and their parents if they are aware of parents' views towards the condition.
